# Sensitivity of Air Pollution-Induced Premature Mortality to Precursor Emissions under the Influence of Climate Change

**DOI:** 10.3390/ijerph7052222

**Published:** 2010-05-05

**Authors:** Efthimios Tagaris, Kuo-Jen Liao, Anthony J. DeLucia, Leland Deck, Praveen Amar, Armistead G. Russell

**Affiliations:** 1 School of Civil and Environmental Engineering, Georgia Institute of Technology, 311 Ferst Drive Atlanta, GA 30332-0512, USA; E-Mails: tagaris@ipta.demokritos.gr (E.T.); kuojenliao@gmail.com (K.-J.L.); 2 Environmental Research Laboratory, NCSR Demokritos, Patriarchou Gregoriou Str. Aghia Paraskevi 153-10, Greece; 3 Department of Surgery, James H. Quillen College of Medicine, East Tennessee State University, Johnson City, TN 37614, USA; E-Mail: DELUCIA@mail.etsu.edu; 4 Stratus Consulting Inc., 1920 L Street, NW; Suite 420, Washington, DC 20036, USA; E-Mail: LDeck@StratusConsulting.com; 5 Northeast States for Coordinated Air Use Management (NESCAUM), 89 South Street, Suite 602, Boston, MA 02111, USA; E-Mail: pamar@nescaum.org

**Keywords:** climate change, premature mortality, ozone, particulate matter, sensitivity, emissions, United States

## Abstract

The relative contributions of PM_2.5_ and ozone precursor emissions to air pollution-related premature mortality modulated by climate change are estimated for the U.S. using sensitivities of air pollutants to precursor emissions and health outcomes for 2001 and 2050. Result suggests that states with high emission rates and significant premature mortality increases induced by PM_2.5_ will substantially benefit in the future from SO_2_, anthropogenic NO_X_ and NH_3_ emissions reductions while states with premature mortality increases induced by O_3_ will benefit mainly from anthropogenic NO_X_ emissions reduction. Much of the increase in premature mortality expected from climate change-induced pollutant increases can be offset by targeting a specific precursor emission in most states based on the modeling approach followed here.

## 1. Introduction

Air pollution can affect human health, having both short-term (e.g., irritation to the eyes, headaches and coughing) and long-term (e.g., chronic respiratory disease and heart disease) effects [1]. Individual reactions to air pollutants depend on the pollutant type, the exposure duration and health status. Mildest effects include increased medication use and subclinical effects while more severe effects include emergency room visits, hospital admissions, and premature mortality. The young, the elderly, diabetics and those with cardiopulmonary disease, such as asthma or severe bronchitis, are the most vulnerable to air pollution exposure. The World Health Organization using population exposure estimates of PM_10_ (particulate matter with aerodynamic diameter less than 10 μm) concentrations in the year 2002 estimates that 865,000 people die prematurely each year from causes directly attributable to outdoor air pollution [2]. The U.S. has the third highest levels worldwide with an estimated 41,200 premature deaths per year, following China (275,600) and India (120,600). In a recent estimate of global burden of disease, outdoor air pollution was estimated to account for about 2% of all cardiopulmonary disease and 1.4% of total premature mortality [3]. Much of the concern stems from ozone (O_3_) and particulate matter (PM). O_3_ exposure decreases lung function, increases airway reactivity, causes lung inflammation, and decreases exercise capacity. Similarly, PM exposure leads to increased rates of respiratory symptoms and illness, decreased lung function, increased asthma exacerbation and also contributes to impaired cardiovascular responses and altered blood coagulation which may precipitate leg and chest pain, heart attacks, stroke, and ultimately premature mortality [e.g., 4–7]. A recent study [8] examining the long-term ozone exposure and mortality found that ozone and PM_2.5_ (particulate matter with aerodynamic diameter less than 2.5 μm) contributed independently to increased annual mortality in U.S. However, there is no significant effect of exposure to ozone on the risk of premature mortality from cardiovascular causes when particulates were taken into account, but there is a significant effect of exposure to ozone on the risk of death from respiratory causes.

Due to their suspected human health effects, significant effort has been made to investigate climate change impacts on O_3_ and PM concentrations [9–11]. Increases in ground-level O_3_ concentrations are expected in the future due, in part, to higher temperatures and more frequent stagnation events, while changes in precipitation will modify PM_2.5_ levels [12]. Further, higher ambient temperatures lead to higher biogenic VOC emissions, so future climate induced emission changes are expected to affect both pollutants’ formation [13]. Mickley *et al.* [14] suggest that the reduced cyclone frequency in a future warmer climate could increase the severity of summertime pollution in the northeastern and Midwestern United States, although the increase of hurricane strength and precipitation might counteract seasonal pollution in some regions [15]. Hogrefe *et al.* [16] estimate that regional climate change alone will increase the summertime daily maximum 8-hour average O_3_ concentration over the eastern United States by 4 ppb in the 2050s. Their results are based on the IPCC A2 emission scenario [17], which is one of the highest future emissions scenarios. Across a number of modeling experiments carried out by different groups, absent accounting for emission decreases to controls, simulated global climate change causes increases of a few to several parts per billion (ppb) in summertime mean maximum daily 8-hour average O_3_ concentrations over substantial regions of the U.S. [18]. The different modeling experiments in general do not, however, simulate the same regional patterns of change. These differences seem to result largely from variations in the simulated patterns of changes in key meteorological drivers, such as temperature and surface insolation.

Bell *et al.* [9] estimated that elevated O_3_ levels would increase daily premature mortality 0.11%–0.27% across 50 U.S. cities in 2050 compared to 2001 based on the IPCC-A2 emissions scenario whereas Knowlton *et al.* found a 4.5% O_3_-related mortality increase [19] in the 31 counties of the New York metropolitan area. It has been also suggested [20] that climate change driven air quality-related health effects will be adversely affected in more than 2/3 of the continental U.S. in 2050 compared to 2001 based on the IPCC-A1B emissions scenario [17]. The IPCC-A1B emissions scenario is one of the business-as-usual emission scenarios which is generally viewed as a midrange case that assumes a future world of rapid economic growth with a balance between fossil and nonfossil energy sources. Although these approaches are used to examine the hypothetical situation of what would happen if the predicted future climate conditions occurred when holding the anthropogenic emission inventory and population constant, the information provided enhances the ability of air quality managers to consider global change in their decisions quantifying the controls that will be needed to meet a given air quality standard (climate penalty). Extending the study by Tagaris *et al.* [20] where the potential health impact of ambient O_3_ and PM_2.5_ concentrations modulated by climate change over the United States has been investigated, in this study we assess the relative contribution of O_3_ and PM_2.5_ precursor emissions in premature mortality change, estimating the sensitivities of premature mortality to emissions and providing an estimate for the emission reductions needed to offset the related mortalities.

## 2. Methods

Results of the Goddard Institute for Space Studies (GISS) Global Climate Model (GCM) [21], and components of the Models-3 atmospheric modeling system [22,23] were used to simulate the impact of climate and emissions changes on air quality. The U.S. EPA’s BENMAP (http://www.epa.gov/air/benmap) is used to translate those air quality changes to health impacts. Details of the modeling approach have been reported elsewhere [13,20,24] and summarized here.

### 2.1. Meteorology

The Fifth-Generation NCAR/Penn State Mesoscale meteorological Model (MM5) [22] is used to downscale (*i.e.*, increase the spatial and temporal resolution over the chosen modeling domain) NASA’s Goddard Institute for Space Studies (GISS) Global Climate Model (GCM) [21] outputs for years 2001 and 2050 [14,25]. The simulation followed the Intergovernmental Panel on Climate Change (IPCC) A1B emission scenario [17] for greenhouse gases. The GISS GCM was applied at a horizontal resolution of 4° latitude by 5° longitude to simulate current and future climate at global scale [14] while the MM5 is applied in a nested configuration with 108 km horizontal resolution for the outer domain and 36 km for the inner one [25]. The inner domain covers the continental United States, part of Canada, Mexico and nearby oceans.

### 2.2. Air Quality Modeling

The Community Multiscale Air Quality model (CMAQ) [23] with the SAPRC-99 [26] chemical mechanism is used to simulate pollutant concentrations (*i.e.*, O_3_ and PM_2.5_) for both historic and future periods keeping constant boundary conditions for 2001 and 2050 simulations [24]. A uniform grid of 36 km × 36 km horizontal cells with 9 vertical layers is employed in the simulations. Although the emission inventory is kept the same (*i.e.*, emission sources, population, activity levels and pollution controls) emissions are not since some pollutant emissions (e.g., biogenic and mobile sources) depend on meteorology. Higher ambient temperatures lead to higher biogenic VOC emissions, suggesting that climate induced emission changes in a warmer environment will affect pollutant formation. The Decoupled Direct Method 3D (DDM-3D) [27–30] is incorporated in the CMAQ to quantify sensitivities of air pollutants to precursor emissions [13]. These sensitivities represent how pollutant concentrations respond to precursor emission changes as if the systems were linear [13]. Although the system is not linear, extensive testing suggests the linear (first-order) response is accurate up to emission changes of the order of 30% for O_3_ and 20–50% for PM_2.5_ (depending on species) [31–33].

### 2.3. Health Effects

The U.S. EPA’s Environmental Benefits Mapping and Analysis Program (BenMAP) ver. 2.4.8 (http://www.epa.gov/air/benmap) was employed to estimate the potential health impact of ambient O_3_ and PM_2.5_ concentration changes due to climate change over the U.S. [20]. BenMAP includes a database of age-specific population and disease incidence rates, and a concentration-response functions library for use in analyzing the health effects driven by changes in air quality. The concentrationresponse functions used are consistent with those in recent regulatory analyses [34–37]. The O_3_ mortality toxicity factor is 0.00052, (*i.e.*, a 1 ppbv change in O_3_ concentrations would lead to a 0.052% change in the expected number of premature deaths) [38] while the PM_2.5_ mortality toxicity factor is 0.0058 (*i.e.*, a 1 μgm^−3^ change in PM_2.5_ concentrations would lead to a 0.58% change in the expected number of premature deaths) [39]. BenMAP does not account for the potential variability in the impacts of different components of PM_2.5_, and the exposure-response estimates are viewed as uncertain and may vary between parts of the country. Here, the default BenMAP ozone-premature mortality relationship is used and is based on 24-hour averaged ozone levels [20]. Since population, mortality rates and disease incidence rates obtained from 2000 are used the anticipated changes in the population (increasing by 2050) and age-specific mortality rates (expected to continue to decrease) would affect future health estimates.

### 2.4. Premature Mortality Sensitivity

In order to estimate, here, the relative contribution of PM_2.5_ and O_3_ precursor emissions in premature mortality changes for each state in the continental U.S. the following formula is used:

$$E_{X(Y)}=\frac{\Delta M_{X}}{\Delta C_{X}}S_{X(Y)}$$

where:

➢ *E_X(Y)_* is the mortality change induced by changes in pollutant *X* concentration due to a 1% reduction in precursor *Y* emissions over the domain
➢ *ΔC_X_* is the pollutant *X* concentration change due to climate change
➢ *ΔM_X_* is the premature mortality change induced by *ΔC_X_*
➢ *S_X(Y)_* is the sensitivity of pollutant *X* to precursor emissions *Y* (*i.e.*, concentration responses to a 1% emissions reduction)
➢ *X*: PM_2.5_ or O_3_ concentrations
➢ *Y*: SO_2_, anthropogenic NO_X_, NH_3_, biogenic or anthropogenic VOC emissions.

Linear responses of the pollutant concentrations, and the resulting changes in premature mortality, to precursor emissions (*i.e.*, how premature mortality would change to a 1% reduction in SO_2_, NOx, NH_3_ or VOC emissions) can be used for emission reductions of up to 25–50%, depending on pollutant and environment, as mentioned above. In this way, the reduction needed in precursor emissions to offset air pollution-induced mortality due to climate change could be estimated for each state. This is the first time, to our knowledge, that an analysis of premature mortality sensitivity to air pollutant precursor emissions is performed.

## 3. Results and Discussion

A detailed discussion of climate change impact modeling results on meteorology and air quality as well as air pollution related health effects have been presented elsewhere [13,14,20,24,25] and key outputs are presented below.

### 3.1. Baseline Meteorology

Temperatures in 2050s are modeled to be higher over the U.S. with an average increase between 1 and 3 degrees [24]. During winter and spring warming is between 0 and 3 degrees. Throughout summer warming between 2 and 4 degrees is simulated over the southwestern U.S. [25]. Warming over the midwestern U.S. is found to be less, while in some regions a small cooling is related to changes in cloud cover. During fall, warming of up to 4 degrees occurs over much of the western U.S. Daily rainfall intensity increases in most regions across the continental U.S., but the change in daily rainfall frequency is more spatially variable. As modeled, changes in rainfall frequency are small during winter and spring [25]. Regional changes in precipitation up to ±5 cm yr^−1^ are simulated for the majority of the states, while in a few states the changes will be higher than ±20 cm yr^−1^ (more rain is simulated in the southeastern states). Extreme positive changes (higher than 50 cm yr^−1^) are simulated over the Atlantic Ocean and Gulf of Mexico [40]. During winter and spring the changes in downward solar radiation is about 8 W/m^2^ in the U.S. [25]. During summer, it reaches 30 W/m^2^ over Texas. In the Midwest, cloud cover changes reduce solar radiation by up to 30 W/m^2^. During fall, the change is positive everywhere, with a maximum over the western U.S. of 15 W/m^2^. The changes in the number of stagnation days during winter and spring are much smaller compared to summer and fall, where the percentage change in stagnation occurrence is very significant [25].

### 3.2. Baseline Air Quality

Climate change modifies mean summer daily maximum 8-hour average O_3_ concentration levels by ±3% and mean annual PM_2.5_ concentrations by −3% to 6% [24]. The lengthening of stagnation events tends to increase summer O_3_ concentrations particularly during intense episodes near cities while a spatially mixed impact on annual PM_2.5_ levels is simulated. The latter effect is mainly due to a variable change in precipitation. Stagnation events are predicted to have the most impact in the west, northeast and plains and a small impact is anticipated in the southeast. Climate change alone leads to increasing O_3_ concentrations in all the examined cities (*i.e.*, Los Angeles, Houston, Chicago, New York, and Atlanta) and more days with daily maximum 8-hour average O_3_ concentration over the air quality standard are predicted in Los Angeles, New York and Houston. First-order (linear) sensitivities suggest [13] that a 10% reduction in anthropogenic NO_X_ emissions causes 2–4% decreases in maximum ozone concentrations. Reductions in VOC emissions are also beneficial for decreasing O_3_ levels. Overall, O_3_ sensitivities to anthropogenic NO_X_, biogenic VOC, and anthropogenic VOC emissions are predicted to increase only slightly in 2050 compared to 2001 due to climate change. SO_2_, NH_3_, anthropogenic NOx and biogenic VOCs were found to be important precursors for PM_2.5_ formation, with climate change modeled to affect slightly these sensitivities.

### 3.3. Baseline Health Effects

Air pollution-related premature mortality will be higher in the future in more than 2/3 of the states due to climate changes. Model results find that New York, along with the states in the Great Lakes and the northeastern U.S. will be affected more. Conversely, Texas and the southeastern states will experience a smaller effect [20]. The PM_2.5_-related health effects dominate the O_3_-related health effects but the geographic pattern of changes in O_3_ concentrations is significantly different than the patterns observed for PM_2.5_. About 4,000 more PM_2.5_-related premature deaths are projected nationally for 2050 compared to 2001 with more incidents in the Great Lakes area and the northeastern U.S. and less in the southern states. In addition, about 300 more O_3_-related premature deaths are projected nationally for 2050 compared to 2001. Climate change-related increased O_3_ health effects are less pronounced in the Great Lakes area and more pronounced for the southern states.

### 3.4. State Specific PM_2.5_, O_3_ and Premature Mortality Sensitivities to Emissions

**Figure 1.**
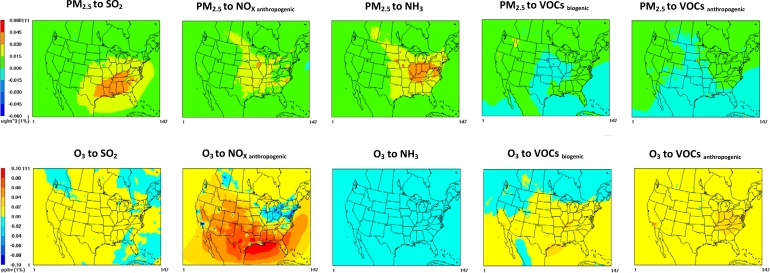
Daily average PM_2.5_ and O_3_ sensitivities* per 1% reduction in domain-wide SO_2_, anthropogenic NO_X_, NH_3_, biogenic or anthropogenic VOC emissions in 2050. * Positive sensitivity (+): Reductions in precursor emissions decrease pollutant concentrations. Negative sensitivity (−): Reductions in precursor emissions increase pollutant concentrations.

PM_2.5_ concentrations are more sensitive to SO_2_, NO_X_ and NH_3_ emissions (Figure 1) than other species (e.g., VOCs). Atmospheric SO_2_ is oxidized to sulfuric acid which reacts with ammonia to form ammonium sulfate while gas-phase NO_X_, oxidizes to nitric acid which reacts with ammonia to form ammonium nitrate [41]. States which are simulated to be more sensitive to SO_2_ emissions are those with elevated SO_2_ emissions such as the eastern states [42] (decreases up to 0.035 μgm^−3^ in daily state average PM_2.5_ concentration for a 1% reduction in SO_2_ emissions), while the western states are less sensitive (decrease between 0.003 and 0.008 μgm^−3^ in daily state average PM_2.5_ concentration for a 1% reduction in SO_2_ emissions). Midwest states are simulated to be more sensitive to anthropogenic NO_X_ emissions since this sub-region experiences relatively large NO_X_ and NH_3_ emissions [42] (decrease up to 0.026 μgm^−3^ in daily state average PM_2.5_ concentration for a 1% reduction in anthropogenic NO_X_ emissions). The sensitivity of PM_2.5_ to NH_3_ emissions follows SO_2_ and NO_X_ spatial distributions and contributes to a decrease of up to 0.04 μgm^−3^ in daily state average PM_2.5_ concentration for a 1% reduction in NH_3_ emissions. The impact of both biogenic and anthropogenic VOC emissions changes to PM_2.5_ concentration is less important. A 1% reduction in VOC emissions decreases daily state average PM_2.5_ concentration in few states (up to 0.01 μgm^−3^ and 0.004 μgm^−3^ for biogenic and anthropogenic emissions, respectively) and increases daily state average PM_2.5_ concentration in other states (up to 0.003 μgm^−3^ and 0.001μgm^−3^ for biogenic and anthropogenic emissions, respectively).

When NO_X_ and VOCs mix in the presence of sunlight, ground level O_3_ is formed [41]. The response of ambient O_3_ formation to reductions in NOx and VOC emissions depends on the relative abundance of NO_X_ and VOCs, as well as meteorological factors. The majority of the states have a positive response to anthropogenic NO_X_ emissions, with a decrease of up to 0.067 ppb in daily state average O_3_ concentration for a 1% emissions reduction. A few states located in the Midwest and Northeast sub-regions have a negative response, with an increase of up to 0.044 ppb in daily state average O_3_ concentration for a 1% reduction in anthropogenic NO_X_ emissions. VOC emissions are also important to O_3_ responses. A 1% reduction in anthropogenic and biogenic VOC emissions are simulated to reduce O_3_ concentrations up to 0.021 ppb in the eastern states while a minor negative response to biogenic VOC emissions is noticed for the northwestern states (an increase of up to 0.005 ppb in daily state average O_3_ concentration for a 1% reduction in biogenic VOC emissions). SO_2_ and NH_3_ emissions are simulated to have a minor impact (a decrease of up to 0.004 ppb and increase of up to 0.006 ppb in daily state average O_3_ concentrations for a 1% reduction in SO_2_ and NH_3_ emissions, respectively).

States with high emission rates [42] and significant premature mortality increases induced by PM_2.5_ concentrations modulated by climate change (*i.e.*, midwestern and northeastern U.S. sub-regions) are estimated to substantially benefit from SO_2_, anthropogenic NO_X_ or NH_3_ emissions reduction (Table 1). Illinois is simulated to be the state where emissions reduction will most significantly decrease PM_2.5_-induced premature mortality: a 1% reduction in SO_2_, anthropogenic NO_X_ or NH_3_ emissions results in 28, 27 and 40 less incidents, respectively. States with fewer related incidents in the future (e.g., Texas, Florida) will also benefit from emissions reduction. In general, reduction in both anthropogenic and biogenic VOC emissions plays a minor role compared to SO_2_, anthropogenic NO_X_ and NH_3_ emissions reduction.

States with premature mortality increases induced by O_3_ concentrations modulated by climate change are estimated to benefit mainly from anthropogenic NO_X_ emissions reduction. In the majority of the states, anthropogenic NO_X_ emissions reduction will reduce premature death, however, in a few states where VOCs are the limiting precursor for O_3_ formation, NO_X_ emissions reductions are found to result in an increase (e.g., NJ, IL, OH, PA, IN). This is partly an artifact of using the exposure-response relationship for O_3_ based on a 24-hour average. 24-hour average O_3_ levels can respond negatively to NO_X_ emissions when 8-hour maximum levels would respond positively. Texas and California are simulated to be the states that will benefit most: a 1% reduction in anthropogenic NO_X_ emissions results in about 4 less premature deaths. Reduction in both anthropogenic and biogenic VOC emissions are also simulated to be beneficial for the states with high premature mortality increase induced by O_3_ concentrations modulated by climate change (between 0.6 and 1.7 fewer incidents for a 1% reduction of VOCs). Northwestern states are simulated to have a small increase in premature mortality due to biogenic VOC emissions reduction. As anticipated, SO_2_ or NH_3_ emissions reductions only slightly modify O_3_-related premature mortality since these two pollutants do not have a large impact on ozone formation.

Generally, the effect of emissions reduction in cumulative (total) premature mortality induced by both PM_2.5_ and O_3_ changes follows the PM_2.5_ trend since PM_2.5_ related mortality has been found higher than that due to O_3_ [20]. In a few states O_3_ related premature mortality modulated by anthropogenic NO_X_ and VOC emissions reduction play an important role in the cumulative results (e.g., NJ and RI for NO_X_, IL, AR, KY and TN for biogenic VOCs, AZ and AL for anthropogenic VOCs) (Table 1).

Reduction in one precursor emission class (*i.e.*, SO_2_, anthropogenic NO_X_, NH_3_, or VOCs) is estimated to be able to offset premature mortality induced by PM_2.5_ and O_3_ changes modulated by climate change in most of the states (Table 2). States with increases of more than 400 premature deaths will be able to offset those incidents by reducing SO_2_ or NH_3_ emissions. For the majority of the states with less than 400 deaths, the reduction in anthropogenic NO_X_ emissions is estimated to be another feasible option to offset the increased premature mortality from climate-related air pollution increases. Reduction in VOC emissions works best in a few states. Nine states (*i.e.*, IL, LA, KY, MS, IA, NM, DE, KS and VT) will be able to offset premature mortality by reducing 17% or less of their SO_2_, anthropogenic NO_X_ or NH_3_ emissions while seven states (*i.e.*, MA, CT, WA, OR, NH, ME, and RI) need reductions in more than one precursor emission class. Although in this study a domain wide emissions reduction has been applied, impacts of precursor emissions on air quality drop quickly with increasing distance between receptor and emission sources [43]. This suggests that emission controls in a specific state will have the major impact in air quality and the induced health effects within that state, except for some of the smaller, downwind states.

## 4. Conclusions

PM_2.5_ and O_3_ induced premature mortality modulated by climate change can be offset in most of the states by reducing only a single precursor emission class (e.g., NO_X_, SO_2_) based on the modeling approach followed here. Reduction in SO_2_ or anthropogenic NO_X_ or NH_3_ emissions is found to be effective in most of the states although in few states VOC emission reductions can be most effective on a percent basis. Combining reductions in more than one pollutant precursor emission class will give synergistic results. As such, the information provided here will enhance the ability of air quality and public health managers to consider global change in their planning, combining the potential impact of climate change on PM_2.5_ and O_3_ - related premature mortalities with PM_2.5_ and O_3_ precursor emissions reduction strategies.

## Figures and Tables

**Table 1.** State specific PM_2.5_, O_3_, and total premature mortality change in 2050 compared to 2001 and the sensitivity per 1% reduction in SO_2_, anthropogenic NO_X_, NH_3_, anthropogenic VOC or biogenic VOC emissions.

	Mortality Change (number of incidents)																	
	Caused by changes in concentrations*			Caused by 1% decrease in SO_2_ emissions			Caused by 1% decrease in anthropogenic NO_x_ emissions			Caused by 1% decrease in NH_3_ emissions			Caused by 1% decrease in biogenic VOC emissions			Caused by 1% decrease in anthropogenic VOC emissions		
	PM_2.5_	O_3_	Total	PM_2.5_	O_3_	Total	PM_2.5_	O_3_	Total	PM_2.5_	O_3_	Total	PM_2.5_	O_3_	Total	PM_2.5_	O_3_	Total
AL	−84	23	−61	−6.55	−0.01	−6.56	−3.26	−1.05	−4.32	−4.70	0.02	−4.68	−1.19	−0.21	−1.40	0.00	−0.30	−0.30
AZ	60	19	79	−3.67	−0.01	−3.67	−2.18	−1.08	−3.25	−2.15	0.01	−2.14	−2.28	−0.18	−2.47	0.00	−0.17	−0.17
AR	−72	21	−51	−6.34	−0.01	−6.35	−4.86	−0.62	−5.47	−5.02	0.02	−5.00	0.12	−0.18	−0.06	0.21	−0.19	0.03
CA	−186	82	−104	−7.44	−0.02	−7.46	−11.35	−4.77	−16.13	−8.42	0.07	−8.35	−13.50	−1.15	−14.64	−0.93	−1.70	−2.63
CO	58	−4	54	−1.40	−0.01	−1.41	−1.07	−1.09	−2.16	−1.45	0.02	−1.43	−0.53	−0.11	−0.64	0.03	−0.09	−0.06
CT	232	−3	229	−2.71	0.00	−2.71	−1.40	0.18	−1.22	−4.25	0.01	−4.24	−0.75	−0.05	−0.80	−0.39	−0.10	−0.48
DE	8	−1	7	−0.87	0.00	−0.87	−0.58	0.03	−0.55	−1.02	0.00	−1.02	−0.04	−0.02	−0.06	−0.09	−0.03	−0.12
DC	2	0	2	−0.09	0.00	−0.09	−0.04	0.00	−0.04	−0.11	0.00	−0.11	−0.01	0.00	−0.01	−0.01	0.00	−0.01
FL	−396	30	−366	−21.96	0.00	−21.96	−5.41	−2.41	−7.81	−10.57	0.03	−10.55	−3.77	−0.50	−4.26	0.18	−0.56	−0.38
GA	−163	34	−129	−8.65	0.00	−8.65	−4.38	−1.24	−5.62	−6.70	0.03	−6.68	−2.10	−0.34	−2.44	−0.10	−0.48	−0.58
ID	23	−5	18	−0.36	0.00	−0.36	−0.50	−0.12	−0.62	−0.49	0.00	−0.49	−1.18	0.02	−1.16	−0.02	−0.01	−0.02
IL	396	−17	379	−28.29	−0.31	−28.60	−27.34	1.19	−26.16	−40.12	0.41	−39.71	1.65	−3.48	−1.83	−1.21	−4.29	−5.50
IN	275	−5	270	−8.94	−0.01	−8.96	−8.05	0.25	−7.80	−13.98	0.02	−13.97	0.28	−0.20	0.07	−0.68	−0.26	−0.94
IA	32	−8	24	−1.98	−0.03	−2.01	−3.39	−0.15	−3.55	−3.56	0.05	−3.51	0.34	−0.12	0.23	0.01	−0.15	−0.14
KS	6	1	7	−0.87	−0.03	−0.90	−0.87	−0.97	−1.84	−0.82	0.05	−0.77	0.11	−0.28	−0.17	0.04	−0.23	−0.19
KY	52	8	60	−15.51	−0.02	−15.54	−7.59	−0.07	−7.66	−18.20	0.02	−18.18	−0.03	−0.31	−0.34	−0.49	−0.36	−0.86
LA	57	32	89	−9.58	−0.01	−9.59	−4.13	−1.01	−5.14	−5.93	0.02	−5.91	−0.95	−0.28	−1.24	0.11	−0.24	−0.13
ME	46	−4	42	−0.67	0.00	−0.67	−0.30	−0.04	−0.34	−0.87	0.00	−0.86	−0.59	0.01	−0.58	−0.05	−0.02	−0.07
MD	90	−3	87	−5.56	0.00	−5.56	−2.79	0.09	−2.70	−6.75	0.00	−6.75	−0.39	−0.05	−0.44	−0.54	−0.08	−0.62
MA	328	−6	322	−4.36	0.00	−4.36	−1.75	0.19	−1.56	−6.22	0.01	−6.20	−1.36	−0.09	−1.45	−0.43	−0.20	−0.63
MI	624	−43	581	−11.81	−0.04	−11.85	−8.71	−0.01	−8.72	−17.20	0.05	−17.15	−2.47	−0.17	−2.64	−0.72	−0.60	−1.32
MN	218	−26	192	−4.75	−0.06	−4.81	−7.58	−0.16	−7.74	−9.78	0.09	−9.70	−1.89	−0.04	−1.93	−0.37	−0.12	−0.49
MS	33	14	47	−11.89	−0.01	−11.90	−6.37	−0.66	−7.02	−8.38	0.01	−8.37	−1.63	−0.12	−1.75	0.15	−0.16	−0.02
MO	−78	19	−59	−25.81	−0.03	−25.83	−24.07	−0.59	−24.66	−28.34	0.04	−28.30	2.38	−0.27	2.10	0.41	−0.32	0.08
MT	16	−4	12	−0.28	0.00	−0.28	−0.22	−0.08	−0.30	−0.40	0.00	−0.40	−0.29	0.01	−0.28	0.00	−0.01	−0.01
NE	−19	−6	−25	−2.08	−0.01	−2.09	−2.40	−0.20	−2.59	−2.71	0.02	−2.69	0.37	−0.05	0.32	0.14	−0.04	0.10
NV	12	1	13	−0.44	0.00	−0.44	−0.38	−0.05	−0.43	−0.45	0.00	−0.45	−0.97	0.00	−0.97	−0.02	0.00	−0.02
NH	60	−2	58	−0.90	0.00	−0.90	−0.46	−0.01	−0.47	−1.43	0.00	−1.43	−0.57	0.00	−0.57	−0.08	−0.03	−0.10
NJ	497	16	513	−11.61	−0.02	−11.63	−6.07	3.94	−2.13	−15.75	0.07	−15.68	−1.72	−1.13	−2.85	−1.75	−1.73	−3.48
NM	16	4	20	−1.91	0.00	−1.92	−1.01	−0.19	−1.20	−1.16	0.00	−1.16	−0.44	−0.02	−0.46	0.06	−0.01	0.05
NY	846	−3	843	−15.07	0.00	−15.07	−10.77	0.02	−10.75	−24.77	0.00	−24.77	−3.50	−0.02	−3.52	−1.15	−0.05	−1.21
NC	−95	9	−86	−8.22	−0.02	−8.24	−4.66	−0.79	−5.45	−7.67	0.05	−7.62	−0.90	−0.66	−1.56	−0.30	−0.92	−1.21
ND	−4	−4	−8	−0.59	0.00	−0.60	−0.79	−0.03	−0.83	−0.95	0.01	−0.94	−0.04	0.00	−0.05	0.01	−0.01	0.00
OH	566	−28	538	−12.55	−0.02	−12.57	−7.73	0.85	−6.87	−20.36	0.04	−20.33	−0.40	−0.48	−0.88	−0.93	−0.70	−1.63
OK	−43	16	−27	−7.82	−0.01	−7.84	−5.97	−0.75	−6.73	−6.00	0.02	−5.98	0.69	−0.18	0.51	0.28	−0.16	0.12
OR	79	−13	66	−1.01	0.00	−1.01	−0.95	−0.26	−1.22	−0.95	0.00	−0.95	−2.78	0.04	−2.74	−0.06	−0.02	−0.08
PA	464	−20	444	−12.56	0.00	−12.56	−7.07	0.38	−6.69	−18.85	0.02	−18.83	−1.30	−0.22	−1.53	−1.08	−0.41	−1.49
RI	43	−1	42	−0.63	0.00	−0.63	−0.22	0.12	−0.11	−0.84	0.00	−0.84	−0.14	−0.04	−0.19	−0.07	−0.08	−0.15
SC	−35	13	−22	−3.56	0.00	−3.56	−2.04	−0.51	−2.55	−3.15	0.02	−3.13	−0.63	−0.26	−0.90	−0.11	−0.35	−0.46
SD	−18	−3	−21	−1.00	−0.01	−1.01	−1.28	−0.08	−1.36	−1.82	0.01	−1.81	0.11	−0.01	0.10	−0.02	−0.01	−0.03
TN	−85	21	−64	−8.33	−0.02	−8.35	−4.11	−0.58	−4.69	−8.08	0.02	−8.06	−0.17	−0.35	−0.52	−0.11	−0.41	−0.52
TX	−536	161	−375	−25.23	−0.05	−25.27	−12.13	−4.03	−16.16	−14.12	0.07	−14.05	1.16	−0.93	0.22	0.88	−0.59	0.28
UT	1	−2	−1	−0.05	0.00	−0.05	−0.05	−0.10	−0.15	−0.06	0.00	−0.06	−0.06	0.00	−0.06	0.00	−0.01	−0.01
VT	7	−2	5	−0.37	0.00	−0.37	−0.32	−0.01	−0.34	−0.71	0.00	−0.70	−0.22	0.00	−0.23	−0.04	−0.02	−0.07
VA	−2	1	−1	−2.88	0.00	−2.88	−1.05	−0.02	−1.07	−3.10	0.00	−3.09	−0.28	−0.06	−0.35	−0.14	−0.10	−0.24
WA	139	−11	128	−1.93	0.00	−1.93	−2.17	−0.18	−2.36	−2.28	0.01	−2.27	−4.42	0.03	−4.39	−0.12	−0.04	−0.16
WV	43	−2	41	−2.73	0.00	−2.73	−0.74	0.01	−0.73	−2.98	0.00	−2.98	−0.20	−0.06	−0.25	−0.11	−0.09	−0.19
WI	196	−18	178	−4.04	−0.02	−4.06	−4.73	−0.05	−4.77	−5.79	0.04	−5.75	−0.69	−0.08	−0.77	−0.19	−0.18	−0.37
WY	2	−2	0	−0.14	0.00	−0.14	−0.07	−0.07	−0.14	−0.17	0.00	−0.17	−0.10	0.00	−0.10	0.00	0.00	−0.01

* Mortality change caused by changes in concentrations has been published in Tagaris *et al.*, 2009 [20].

**Table 2.** Individual precursor emissions reduction needed relative to 2001 emissions to offset cumulative premature mortality induced by PM_2.5_ and O_3_ changes modulated by climate change.

	State*	Premature mortality**	Domain−wide emissions change (%)				
			SO_2_	anthropogenic NO_X_	NH_3_	biogenic VOCs	anthropogenic VOCs

1	NY	843	−56	>60	−34	>60	>60

2	MI	581	−49	>60	−34	>60	>60

3	OH	538	−43	>60	−26	>60	>60

4	NJ	513	−44	>60	−33	>60	>60

5	PA	444	−35	>60	−24	>60	>60

6	IL	379	−13	−14	−10	>60	>60

7	MA	322	>60	>60	−52	>60	>60

8	IN	270	−30	−35	−19	−	>60

9	CT	229	>60	>60	−54	>60	>60

10	MN	192	−40	−25	−20	>60	>60

11	WI	178	−44	−37	−31	>60	>60

12	WA	128	>60	−54	−56	−29	>60

13	LA	89	−9	−17	−15	>60	>60

14	MD	87	−16	−32	−13	>60	>60

15	AZ	79	−22	−24	−37	−32	>60

16	OR	66	>60	−54	>60	−24	>60

17	KY	60	−4	−8	−3	>60	>60

18	NH	58	>60	>60	−41	>60	>60

19	CO	54	−38	−25	−38	>60	>60

20	MS	47	−4	−7	−6	−27	>60

21	ME	42	>60	>60	−49	>60	>60

22	RI	42	>60	>60	−50	>60	>60

23	WV	41	−15	−56	−14	>60	>60

24	IA	24	−12	−7	−7	−	>60

25	NM	20	−10	−17	−17	−43	−

26	ID	18	−50	−29	−37	−16	>60

27	NV	13	−29	−30	−29	−13	>60

28	MT	12	−43	−40	−30	−42	>60

29	DE	7	−8	−13	−7	>60	−57

30	KS	7	−8	−4	−9	−40	−36

31	VT	5	−14	−15	−7	−22	>60

32	DC	2	−23	−49	−18	>60	>60

* States with premature mortality increase;

** Premature mortality change has been published in Tagaris *et al.*, 2009 [20].

## References

[1] Curtis L Rea W Smith-Willis P Fenyves E Pan Y Adverse health effects of outdoor air pollutants Environ. Int 2006 32 815 830 16730796 10.1016/j.envint.2006.03.012

[2] World Health Organization Estimated Deaths & DALYs Attributable to Selected Environmental Risk Factors 2007 Available online: www.who.int/quantifying_ehimpacts/countryprofilesebd.xls (accessed on 20 April 2010)

[3] Ostro B Outdoor Air Pollution: Assessing the Environmental Burden of Disease at National and Local LevelsWHO Environmental Burden of Disease Series, No 5, World Health Organization Geneva, Switzerland 2004 viii

[4] Bernard SM Samet JM Grambsch A Ebi KL Romieu I The potential impacts of climate variability and change on air pollution-related health effects in the United States Environ. Health Perspect 2001 109 199 209 11359687 10.1289/ehp.109-1240667 PMC1240667

[5] Peng RD Dominici F Pastor-Barriuso R Zeger SL Samet JM Seasonal analyses of air pollution and mortality in 100 US cities Am. J. Epidemiol 2005 161 585 594 15746475 10.1093/aje/kwi075

[6] Ballester F Medina S Boldo E Goodman P Neuberger M Iniguez C Kunzli N Reducing ambient levels of fine particulates could substantially improve health: A mortality impact assessment for 26 European cities J. Epidemiol. Community Health 2008 62 98 105 18192596 10.1136/jech.2007.059857

[7] Araujo JA Barajas B Kleinman M Wang XP Bennett BJ Gong KW Navad M Harkema J Sioutas C Lusis AJ Nel AE Ambient particulate pollutants in the ultrafine range promote early atherosclerosis and systemic oxidative stress Circ. Res 2008 102 589 596 18202315 10.1161/CIRCRESAHA.107.164970 PMC3014059

[8] Jerrett M Burnett RT Pope CA Ito K Thurston G Krewski D Shi Y Calle E Thun M Long-Term Ozone Exposure and Mortality N. Engl. J. Med 2009 360 1085 1095 19279340 10.1056/NEJMoa0803894 PMC4105969

[9] Bell ML Goldberg R Hogrefe C Kinney PL Knowlton K Lynn B Rosenthal J Rosenzweig C Patz JA Climate change, ambient ozone, and health in 50 US cities Clim. Change 2007 82 61 76

[10] Ebi KL Paulson JA Climate change and children Pediatr. Clin. N. Am 2007 54 213 226 10.1016/j.pcl.2007.01.004 17448357

[11] Ebi KL McGregor G Climate change, tropospheric ozone and particulate matter, and health impacts Environ. Health Perspect 2008 116 1449 1455 19057695 10.1289/ehp.11463 PMC2592262

[12] Jacob DJ Winner DA Effect of climate change on air quality Atmos. Environ 2009 43 51 63

[13] Liao K-J Tagaris E Manomaiphiboon K Napelenok SL Woo J-H He S Amar P Russell AG Sensitivity of ozone and fine particulate matter formation to emissions under the impact of potential future climate change Environ. Sci. Technol 2007 41 8355 8361 18200863 10.1021/es070998z

[14] Mickley LJ Jacobs DJ Field BD Rind D Effects of future climate change on regional air pollution episodes in the United States Geophys. Res. Lett 2004 31 L24103

[15] Webster PJ Holland GJ Curry LA Chang HR Changes in tropical cyclone number, duration and intensity in a warming environment Science 2005 309 1844 1846 16166514 10.1126/science.1116448

[16] Hogrefe C Lynn B Civerolo K Ku J-Y Rosenthal J Rosenzweig C Goldberg R Gaffin S Knowlton K Kinney PL Simulating changes in regional air pollution over the eastern United States due to changes in global and regional climate and emissions J. Geophys. Res 2004 109 D22301

[17] Intergovernmental Panel on Climate Change (IPCC) Special Report on Emissions Scenarios: A Special Report of Working Group III of the Intergovernmental Panel on Climate Change Nakićenović N Swart R Cambridge University Press New York, NY, USA 2000

[18] Weaver CP Liang X-Z Zhu J Adams PJ Amar P Avise J Caughey M Chen J Cohen RC Cooter E Dawson JP Gilliam R Gilliland A Goldstein AH Grambsch A Guenther A Harley RA He S Hemming B Hogrefe C Huang H-C Hunt S Jacob D Kinney P Kunkel K Lamarque J-F Lamb B Larkin N Leung LR Liao K-J Lin J Lynn BH Manomaiphiboon K Mass C McKenzie D Mickley L O’Neil S Nolte C Pandis SN Racherla PN Rosenzweig C Russell AG Salathe E Steiner AL Tagaris E Tao Z Wiedinmyer C Williams A Winner D Woo J-H Wu S Wuebbles DJ A preliminary synthesis of modeled climate change impacts on U.S. regional ozone concentrations Bull. Am. Meteorol. Soc 2009 90 1843 1863

[19] Knowlton K Rosenthal JE Hogrefe C Lynn B Gaffin S Goldberg R Rosenzweig C Civerolo K Ku J-Y Kinney PL Assessing ozone-related health impacts under a changing Climate Environ. Health Perspect 2004 112 1557 1563 10.1289/ehp.7163 PMC1247621 15531442

[20] Tagaris E Liao K-J DeLucia AJ Deck L Amar P Russell AG Potential impact of climate change on air pollution-related human health effects Environ. Sci. Technol 2009 43 4979 4988 19673295 10.1021/es803650w

[21] Rind D Lerner J Shah K Suozzo R Use of on line tracers as a diagnostic tool in general circulation model development: 2. Transport between the troposphere and the stratosphere J. Geophys. Res. Atmos 1999 104 9151 9167

[22] Grell G Dudhia J Stauffer DR A Description of The Fifth Generation Penn State/NCAR Mesoscale Model (MM5)NCAR Tech. Note, NCAR/TN-398+STR, Natl. Cent for Atmos. Res.: Boulder, CO, USA,1994

[23] Byun D Schere KL Review of the governing equations, computational algorithms, and other components of the Models-3 Community Multscale Air Quality (CMAQ) modeling system Appl. Mech. Rev 2006 59 51 77

[24] Tagaris E Manomaiphiboon K Liao K-J Leung L-R Woo J-H He S Amar P Russell AG Impacts of global climate change and emissions on regional ozone and fine particulate matter concentrations over the United States J. Geophys. Res. Atmos 2007 112 D14312

[25] Leung LR Gustafson WI Potential regional climate change and implications to US air quality Geophys. Res. Lett 2005 32 L16711

[26] Carter WPL Documentation of the SAPRC-99 Chemical Mechanism for VOC Reactivity AssessmentFinal Report to CA Air Resources Board, contract no. 92–329, and 95–3082000

[27] Dunker AM Efficient calculation of sensitivity coefficients for complex atmospheric models Atmos. Environ 1981 15 1155 1161

[28] Dunker AM The decoupled direct method for calculating sensitivity coefficients in chemical-kinetics J. Chem. Phys 1984 81 2385 2393

[29] Yang YJ Wilkinson JG Russell AG Fast, direct sensitivity analysis of multidimensional photochemical models Environ. Sci. Technol 1997 31 2859 2868

[30] Dunker AM Yarwood G Ortmann JP Wilson GM The decoupled direct method for sensitivity analysis in a three dimensional air quality model—Implementation, accuracy, and efficiency Environ. Sci. Technol 2002 36 2965 2976 12144274 10.1021/es0112691

[31] Hakami A Odman MT Russell AG Nonlinearity in atmospheric response: A direct sensitivity analysis approach J. Geophys. Res. Atmos 2004 109 D15303

[32] Cohan DS Hakami A Hu YT Russell AG Nonlinear response of ozone to emissions: Source apportionment and sensitivity analysis Environ. Sci. Technol 2005 39 6739 6748 16190234 10.1021/es048664m

[33] Napelenok SL Cohan DS Hu YT Russell AG Decoupled direct 3D sensitivity analysis for particulate matter (DDM-3D/PM) Atmos. Environ 2006 40 6112 6121

[34] U. S. EPA Air Quality Criteria for Particulate MatterEPA/600/P-99/002aF; Washington, DC, USA, 2004

[35] U.S. EPA Air Quality Criteria for Ozone and Related Photochemical OxidantsEPA600/R-05/004aF; Washington, DC, USA, 2006

[36] U.S. EPA Final Regulatory Impact Analysis: PM_2.5_ NAAQSPrepared by Office of Air and Radiation, 2006; Available online: http://www.epa.gov/ttn/ecas/ria.html (accessed on 20 April 2010).

[37] U. S. EPA Final Ozone NAAQS Regulatory Impact AnalysisPrepared by Office of Air and Radiation, 2008; Available online: http://www.epa.gov/ttn/ecas/ria.html (accessed on 20 April 2010).

[38] Bell ML McDermott A Zeger SL Samet JM Dominici F Ozone and short-term mortality in 95 US urban communities,1987–2000 J. Am. Med. Assoc 2004 292 2372 2378 10.1001/jama.292.19.2372 PMC3546819 15547165

[39] Pope CA Burnett RT Thun MJ Calle EE Krewski D Ito K Thurston GD Lung cancer, cardiopulmonary mortality, and long-term exposure to fine particulate air pollution J. Am. Med. Assoc 2002 287 1132 1141 10.1001/jama.287.9.1132 PMC4037163 11879110

[40] Tagaris E Liao K-J Manomaiphiboon K Woo J-H He S Amar P Russell AG Impacts of future climate change and emissions reductions on nitrogen and sulfur deposition over the United States Geophys. Res. Lett 2008 35 L08811

[41] Seinfeld J Pandis SN Atmospheric Chemistry and Physics John Wiley Hoboken, NJ, USA 1998

[42] Woo J-H He S Tagaris E Liao K-J Manomaiphiboon K Amar P Russell AG Development of North American emission inventories for air quality modeling under climate change J. Air Waste Manage. Assoc 2008 58 1483 1494 10.3155/1047-3289.58.11.1483 19044164

[43] Napelenok SL Flabermacher FD Akhtar F Hu Y Russell AG Area of influence (AOI) sensitivity analysis: Application to Atlanta, Georgia Atmos. Environ 2007 41 5605 5617

